# Updated recommendations for the design of therapeutic trials for neonatal seizures

**DOI:** 10.1038/s41390-025-04735-1

**Published:** 2026-01-21

**Authors:** Janet S. Soul, Sonya Wang, Cia Sharpe, Betsy Pilon, Ronit M. Pressler, Marilee C. Allen, Fahimeda Ali, Stephane Auvin, Christine Barry, Scott Denne, Hannah C. Glass, Agnes V. Klein, Neil Marlow, Heike Rabe, Kanwaljit Singh, Collin Hovinga

**Affiliations:** 1 Department of Neurology, Boston Children’s Hospital and Harvard Medical School, Boston, MA USA; 2 Department of Neurology and Pediatrics, University of Minnesota School of Medicine, Minneapolis, MN USA; 3 Department of Paediatric Neurology, Starship Children’s Hospital, Auckland, New Zealand; 4 Department of Neurosciences, University of California, San Diego, CA USA; 5 Hope for HIE, MI West Bloomfield, USA; 6 Clinical Neuroscience, UCL GOS Institute of Child Health and Department of Clinical Neurophysiology, Great Ormond Street Hospital for Children NHS Foundation Trust, London, UK; 7 Department of Pediatrics, Johns Hopkins School of Medicine, Infant Neurodevelopmental Center, Kennedy Krieger Institute, Baltimore, MD USA; 8 Paediatric Unit, Innovative Medicines, Healthcare Quality & Access, MHRA, London, UK; 9 Pediatric Neurology Department, Reference Center for Rare Epilepsies, Member of ERN Epicare, Hôpital Universitaire Robert Debré, Paris, France; 10 Institut Hospitalo-Universitaire Robert-Debré du Cerveau de l’Enfant, Paris, France; 11 Université Paris-Cité, INSERM NeuroDiderot, Paris, France; 12 Institut Universitaire de France (IUF), Paris, France; 13 The Critical Path Institute, Tucson, AZ USA; 14 Department of Pediatrics, Indiana University School of Medicine, Indianapolis, IN USA; 15 Department of Neurology and Weill Institute for Neurosciences, Department of Pediatrics, and Department of Epidemiology & Biostatistics, University of California, San Francisco, CA USA; 16 Health Canada, Toronto, ON Canada; 17 UCL EGA Institute for Women’s Health, University College London, London, UK; 18 Department of Academic Paediatrics, Brighton and Sussex Medical School, University of Brighton and University of Sussex, Brighton, UK; 19 Department of Neonatology, University Hospitals Sussex NHS Foundation Trust, Royal Sussex County Hospital, Brighton, UK

## Abstract

**Abstract:**

The International Neonatal Consortium Seizure Working Group of the Critical Path Institute provides an update to the original recommendations for design of clinical trials to treat neonatal seizures based on recent experiences from several trials and developments in the field. Although there aren’t sufficient new data to inform definitions of optimal efficacy endpoints, the Working Group recommended inclusion of alternate measures of seizure burden reduction as secondary or exploratory endpoints, to elucidate clinically meaningful efficacy endpoints for future trials. It was recommended to include additional key covariates, such as timing of seizure onset/cessation, randomization, and ASM administration. There are new recommendations regarding potential for unmasked or single-masked trials, and reporting of concomitant medications and adverse events, and genetic testing. Importantly, specific recommendations were added regarding improved strategies for recruitment and consent, including the use of novel technologies and the involvement of patient advocacy groups. Recommendations regarding trial infrastructure and operational feasibility were included to facilitate trial initiation and conduct, given the many logistical challenges of conducting neonatal seizure treatment trials. Finally, the recommendations consider accommodations for local or national regulations and resources, to ensure that trials are conducted as appropriate to the setting in which the patients are treated.

**Impact:**

- This review provides an update to the initial recommendations for neonatal seizure treatment trial design, with recommendations regarding: (1) exploratory endpoints to inform future trials, (2) additional key covariates to include, (3) considerations regarding masking of investigators, (3) inclusion of concomitant medications and adverse events, (4) genetic testing, (5) strategies for recruitment and consent, and (6) infrastructure and operational feasibility.

## Introduction

Seizures are one of the most common neurological emergencies in the neonatal intensive care unit (NICU) and are associated with significant morbidity and mortality in affected neonates. The large majority of these are acute symptomatic seizures associated with acquired brain injury due to neonatal encephalopathy (NE), or vascular insults such as ischemic stroke or intracranial hemorrhage.^1^ The management of neonatal seizures is a major challenge for clinicians because of subtle or even absent clinical signs, the need for continuous, conventional video-electroencephalography (cvEEG)-based diagnosis and the relatively limited response to anti-seizure medications (ASMs).

Phenobarbital was recently approved by the FDA for the treatment of neonatal seizures and remains the first-line ASM in neonates.^1–4^ Despite long-standing and widespread use of phenobarbital in clinical practice, reported efficacy is highly variable, with seizure cessation rates ranging ~40–80% across reported trials and observational studies,^5–7^ and short-term adverse effects such as respiratory depression are frequent. There is an urgent need for novel drug development programs that incorporate data from randomized controlled trials (RCT) to test the safety and efficacy of new ASMs in neonates.

The Critical Path Institute (C-Path) International Neonatal Consortium (INC) Seizure Working Group previously reported consensus-based recommendations for the design of therapeutic trials for neonatal seizures.^8^ This work resulted from a collaboration of key stakeholders from research institutions, the pharmaceutical industry, regulatory agencies, nursing groups, and patient/family advocacy, and aimed to promote more successful and efficient trials of ASMs for this vulnerable population. Since that time, several recent clinical trials have been conducted evaluating ASMs for neonatal seizures,^6,9,10^, sometimes citing INC recommendations in the development of trial design. However, there are persistent challenges with effective recruitment and trial feasibility,^10^ while the clinical development of new ASMs and optimization of existing ASM use for neonates remains an unmet clinical need in the NICU.

To address this gap, the INC Seizure Working Group was reconvened to evaluate the experience from recent neonatal seizure trials and to develop and refine consensus-based recommendations to address identified challenges with trial recruitment and feasibility and update other aspects of trial design and conduct. Using similar methodology (described in the Working Group’s original recommendations^8^), a modified Delphi process was applied including agreement on key areas for recommendations, formulation of statements for the recommendations around the agreed key areas (including review of relevant legislation/guidance documents from regulatory authorities and recent consensus-based guidelines from academic professional societies), followed by an iterative process of amendment and agreement within working subgroups before all working group stakeholders provided further feedback and a final consensus was reached. This report summarizes the working group’s findings and recommendations for future neonatal seizure trials, addressing challenges and opportunities related to trial design, endpoints, recruitment/consent, and operational feasibility.

## Trial design

### Treatment arms

The Working Group agreed that the recommendations regarding treatment arms should remain unchanged.^8^ That is, there is continued agreement that head-to-head comparisons of ASMs using an active control parallel study design would be needed from a regulatory perspective for demonstrating efficacy in Phase 3 trials, given ethical concerns related to placebo-controlled designs. An active comparator group is needed for Phase 2 trials to establish safety and ASM response in newborns who have associated critical illness, multi-system organ dysfunction or medical disorders specific to one or more seizure etiologies. Some early phase pharmacokinetic studies could be single arm, with add-on of the new ASM to standard therapy to avoid a placebo design.

### Comparator

Similarly, the current working group agreed that phenobarbital remains the standard comparator for Phase 3 trials,^8^ given that phenobarbital is the ASM most used as first-line therapy and is now the single ASM approved by the FDA to treat neonatal seizures. Whether the trial is designed to evaluate superiority or noninferiority to phenobarbital with regard to efficacy or safety profile will depend upon the characteristics and expected clinical effects of the specific ASM being evaluated. Historical controls would not be suitable, because of lack of detailed information about timing and frequency of seizures and ASM administration in most prior studies, differences in patient populations, study eligibility, EEG monitoring strategies/timing and study design.

### Blinding/masking in neonatal seizure trials

Double masking of treatment assignment is the gold standard recommended for RCTs of ASMs in neonatal seizures, as was recommended in the original publication. For injectable medications, masking involves sterile compounding of the ASM being tested as well as masking the control product. However, this complex and costly process is not always feasible in investigator-initiated studies. In addition, because of the clinically evident effects of phenobarbital on the neonate’s level of consciousness, respiration, and EEG, it may not be feasible to truly mask the treatment team, depending on the type of ASM being compared with phenobarbital. Therefore, it may be ethical and reasonable to conduct unmasked or single-masked trials. To offset these shortcomings, efforts to avoid bias must be rigorous. Review of EEG data to verify seizure presence and treatment success by an independent neurophysiologist with neonatal EEG expertise who is masked to treatment assignment is recommended to decrease bias. The assessors of long-term neurodevelopmental outcomes should also remain masked to the treatment arm, even if the treatment arms are unmasked to other study investigators.

### Antiseizure medication considerations

#### Formulations

New ASMs should be developed as age-appropriate formulations with suitable dosage forms and infusion rates to minimize the risk of fluid overload. Formulations should also consider avoidance of excipients which might have short- and long-term adverse effects in newborns. ASM formulations were discussed in detail in the Supplement of the previous consensus-based recommendations.^8^

#### Dose escalation logistics

A dose escalation or dose finding trial requires scientific justification of the dose escalation with consideration of preclinical, pharmacological and all preliminary clinical data (including dose-response modeling and simulation studies) regarding ASM efficacy and safety. There should be evaluation of the pharmacokinetics of a second ASM dose at minimum, to evaluate accumulation, if more than one ASM dose is planned for clinical treatment or a Phase 3 trial. Steady state does not need to be evaluated with a multidose study for acute neonatal seizures since chronic dosing is not typically employed, but may be needed or considered for ASM trials for neonatal onset epilepsy.

#### ASM pharmacokinetics (PK), pharmacodynamics (PD) and dose exposure

The ASM levels and exposure-response with EEG-proven seizures should be measured and reported for ASMs being tested in a trial, at all doses administered if multiple doses are tested.^9–11^ The need for multiple ASM concentrations to determine PK and dose exposure requires low volume samples (particularly for preterm neonates), such as might be collected by dried spot analysis, and sensitive measurement techniques, such as liquid chromatography–tandem mass spectrometry (LC-MS-MS).^12^ Therapeutic hypothermia can impact PK and drug exposure thus needs to be incorporated in the PK/PD analysis. Modeling and simulation approaches, such as population PK, exposure-response, and physiologically based PK, can optimize the initial PK and dose strategy and confirm the final dose recommendation.

#### Concomitant medications

All concomitant medications administered to participants in an ASM treatment trial should be recorded and reported in trial publications. This is required from a regulatory perspective to assess potential effect(s) of concomitant medications on PK of the trial ASM and any interactions with that or other ASMs. All ASMs used in addition to the trial ASM must be reported. It might be pertinent to report maternal medications administered during the pregnancy or at least labor and delivery to evaluate potential interactions for ASMs administered during the first week after birth.

### Genetic testing

Monogenic epilepsy is the most common etiology for neonatal-onset epilepsy with a normal brain MRI. Where feasible, genetic testing is strongly encouraged for treatment trials, ideally with rapid whole genome sequencing in the NICU. Whole exome sequencing or targeted gene panels for neonatal epilepsy/seizures remain reasonable alternatives. Genetic diagnosis is key for patient stratification and analysis of treatment response (may be post-hoc analysis), as genes such as KCNQ2 and SCN2A predict sensitivity to sodium channel blockers, while others might indicate drug resistance.^2,13^ Neonates enrolled in a trial may need to be excluded if or when a suspected or confirmed genetic epilepsy requires precision treatment not included in the trial protocol. Pharmacogenomic variants still need to be identified to better understand drug metabolism and therapy choices.

Genetic testing introduces challenges in consent, intellectual property, and data access. The complexity of genomic findings and their implications place a burden on families, requiring robust informed consent and genetic counseling. Ownership of genetic data, particularly in industry-sponsored trials, must be transparent, balancing research advancement with patient rights. Data sharing should comply with privacy regulations, ensuring families have access to clinically relevant results while maintaining confidentiality. Ethical concerns include the long-term use of neonatal genomic data.^14^ Since newborns cannot consent, policies must address future data use and patient autonomy as they mature.

### Adverse events

Any trial of ASMs for neonatal seizures, including early phase trials, should report adverse events (AEs), including serious AEs as defined by guidelines for Good Clinical Practice.^15^ As described in the first publication, estimated rates of AEs should be defined prior to trial commencement, according to the expected population of neonates. Since that publication, INC developed and validated a neonatal AE severity scale to help researchers use a common approach when reporting AEs, which can be used in future trials.^16–19^ INC collaborated with the Medical Dictionary for Regulatory Activities (MedDRA) Maintenance and Support Services Organization (MSSO) to enhance the INC AE Terminology by providing a mapping of the INC AEs to Medical MedDRA lowest level terms. Standard reference laboratory values, such as those provided by the National Institute of Allergy and Infectious Diseases, should be used consistently to determine the severity of AEs.

### Statistical considerations, including covariates

Statistical considerations for the trial design and analysis plan include those outlined in the original publication, including the need to minimize sample size for the sake of trial feasibility, while taking into account the significant challenges of analyzing safety and efficacy and including predefined expected effect size (for Phase 3 trials), setting appropriate noninferiority margins (if applicable), sensitivity/subgroup analyses, and multiple key covariates, including covariates that may not be equally balanced among treatment groups.^9^

#### Covariates

Neonatal seizure treatment trials should record, analyze, and report key covariates that inform ASM safety and efficacy. The original working group’s publication included a description of multiple covariates that should be integrated into the trial design and statistical analysis plan. As described previously, important covariates to incorporate into the design and analysis plan include seizure etiology, severity of encephalopathy (for NE, graded with a standard scoring system), and pre-treatment EEG background pattern.^8^ Seizure severity or seizure burden remains a critically important covariate, but there still is not agreement on the best measure of seizure severity, since it is not yet established which measure(s) of seizure burden are most important with regard to epileptogenesis and long-term neurologic outcome. Status epilepticus should be included in the measurement of seizure severity. Notably, the lack of a universally accepted definition of duration of a seizure and what constitutes neonatal status epilepticus is challenging for trial conduct and analyses. Since status epilepticus is not yet clearly defined for neonates,^20–23^ the protocol should include the definition used in the trial with scientific rationale, and include the definition of a seizure (e.g., with/without a minimum duration). Note that at the time of writing, the International League Against Epilepsy (ILAE) neonatal task force is working to define neonatal status epilepticus.^20^ Timing of seizure onset and cessation (for acute symptomatic seizures that resolve within hours to days), timing of randomization and trial ASM administration also should be included, since experimental and clinical studies have demonstrated the relationship between timing of seizure treatment and efficacy of that treatment,^5,24–26^ as occurs in older children.^27^ Finally, as described previously, treatment with hypothermia^26^ or other therapies that may affect ASM safety, pharmacokinetics and/or efficacy should be reported and analyzed, together with clinical characteristics relevant to the particular neonatal population and ASM tested.

## Endpoints/neonatal seizure outcome measures

The working group reviewed original study endpoint recommendations based on the previously conducted literature review of neonatal seizure trials and the international survey of neonatal seizure treatment practice.^8^ Additionally, the working group reviewed the endpoints evaluated in neonatal seizure trials^6,9,10,28,29^ reported following the publication of the original recommendations as well as recent consensus guidelines from the Neonatal Task Force of the ILAE.^2,30^

There was continued agreement with the original Working Group recommendations that the primary endpoint of ASM efficacy should remain a predefined measure of seizure reduction or cessation given the primary mechanism of action and intended use of an ASM.^8^ While there was agreement on the importance of long-term neurodevelopmental outcome as a secondary efficacy and safety endpoint, a clinically meaningful reduction in seizure burden remains the recommended primary endpoint to demonstrate ASM efficacy for regulatory decision making. Given the unmet clinical need, some working group members noted it may be reasonable to evaluate comprehensive long-term neurodevelopmental safety via post-marketing requirements to expedite availability of effective therapies if robust efficacy (i.e., reduction of neonatal seizure burden) and intermediate-term safety are demonstrated. Registries might also be considered as supportive data. Notably, seizure etiology needs to be accounted for in any analysis of long-term neurodevelopmental outcome, given the relationship between primary seizure etiology and neurodevelopmental outcome, e.g., for NE vs. stroke vs. brain malformation.

At the time of writing, there are no new nonclinical or clinical data to inform an evidence-based definition of “clinically meaningful” reduction in seizure burden. Therefore, the working group continues to acknowledge that a “clinically meaningful” reduction in seizure burden remains largely consensus-based and is conceptually founded upon the notion that reducing neonatal seizure burden may reduce epileptogenesis and seizure-associated brain injury (or exacerbation of hypoxic-ischemic injury) manifesting as later neurodevelopmental impairment and/or epilepsy. Consistent with this notion, the ILAE guidelines^2^ recommend treatment of neonatal seizures (including electrographic-only seizures) to reduce seizure burden, which may be associated with improved neurologic outcomes (neurodevelopment, reduced rates/severity of later epilepsy). To inform this recommendation, the ILAE Neonatal Task Force conducted a systematic review that identified limited clinical data from 2 RCTs evaluating alternate approaches to seizure detection and management^31,32^ (both underpowered to assess impact of seizure reduction on neurodevelopmental outcomes) and from observational studies^1,33–39^ linking higher seizure burden with adverse neurodevelopmental outcomes. Given the lack of evidence from RCTs to inform whether clinical efforts to reduce seizure burden improve outcomes, the ILAE recommendation is largely based on an expert Delphi-based consensus that concluded with moderate agreement (74% of respondents completely or mostly agreed) that treatment of neonatal seizures may be associated with improved neurodevelopmental and epilepsy outcomes. More robust evidence from future clinical trials would be vital to support recommendations regarding the benefit (or harm) of neonatal seizure treatment than moderate agreement by Delphi-based consensus.

There was general continued agreement with the original working group’s proposed approach to measuring seizure burden including a baseline observation of at least 30 s/h of electrographic seizure activity confirmed by neonatal EEG expert; study drug administered ideally within 30 min (maximum 2 h) of meeting baseline eligibility burden; and measurement of seizure response for at least a 2 h period following time of expected peak drug exposure. While there was general agreement with the previously conducted international survey’s finding of ≥80% reduction in seizure burden as the most commonly cited optimal drug response, other proposed clinically relevant efficacy endpoints included complete seizure cessation for a 24-h period, resolution of status epilepticus (if present), and need for rescue ASM. There were 5 published reports of clinical trials evaluating ASMs in neonates since 2019. Two trials were early phase trials evaluating PK and safety of bumetanide^9^ and brivaracetam,^10^ the bumetanide RCT also evaluated bumetanide exposure-response,^9^ and 4 RCTs compared effectiveness of levetiracetam versus phenobarbital for neonatal seizures.^6,28,29,40^ Three RCTs were conducted with assessment of efficacy based on EEG-determined seizure burden,^6,9,10^ and three other trials were conducted in low-resource settings and evaluated cessation of *clinical* seizures for at least 24 h after ASM loading dose administration.^28,29,40^ Cessation of clinical seizures is not considered sufficient to demonstrate ASM efficacy, given high rates of electrographic-only seizures, particularly after treatment with phenobarbital. The NeoLEV2 study^6^ was a phase IIb RCT that evaluated a primary outcome of complete seizure freedom for 24 h, assessed by independent review of EEGs by 2 neurophysiologists.

There was discussion of consideration of novel endpoints such as achieving cumulative seizure thresholds below what might be considered clinically relevant thresholds (e.g., 40–60 min^37,41^) or reducing maximum hourly seizure burden^42^ from pretreatment levels. It was noted, however, that seizure etiology may be an important determinant of the relevant threshold for these measures, thus, etiology should be included as a key covariate when analyzing seizure burden endpoints.^43^ It was also recommended that future trials include such novel alternate measures of seizure burden reduction as secondary endpoints to inform future trials. For early phase trials, there was continued agreement with acceptance of a smaller reduction in seizure burden (e.g., 30%), adjusting for pre-treatment or total seizure burden if needed, to account for smaller sample sizes and early assessment of ASM response to select agents to test in larger efficacy trials.

## Recruitment/consent

While insufficient recruitment or enrollment are primary reasons for early termination of clinical trials in general,^44^ these challenges are intensified in the NICU by the vulnerability of the population, complexity/high acuity of the clinical condition, high parental stress, and short time windows to achieve effective informed consent.^45–49^ The original working group recommendations included discussion of ethical considerations, the need for full written consent with parental signature, consideration of a continuous consenting process and patient/family and public involvement in trial design and implementation.^8^ While these are still important, a greater emphasis on discussion of the recruitment and consent process was recommended, given that this was a primary issue cited for slow recruitment and/or early termination of prior neonatal seizure trials.^9,10,50–54^ Additionally, it was noted that the recent revised Declaration of Helsinki^55^ emphasizes that vulnerable groups can also be harmed by *not* being including in medical research, as this could perpetuate or exacerbate disparities. Thus, the discussion focused on the need for balance between overly restrictive protections and the need to optimize feasibility and inclusion in trials.

### Alternative consent approaches

Although many neonatal trials, including trials of acute neurological interventions^56–59^ and neonatal seizure treatment^6,9^ have been completed using a traditional full written consent process with parental signature (often with the aid of the study hospital’s transport team and/or facilitated by use of phone/fax/secure email communication), there was extensive discussion regarding alternative approaches to consent. Specifically, there was consideration of the potential acceptability of “exceptions for informed consent” (EFIC US terminology) or “deferred consent” (terminology used elsewhere) in neonatal seizure trials. Regulations in the US,^60,61^ EU^62^, and Japan^63^ regarding consent for clinical trials in emergency situations were reviewed. Requirements are similar across regulatory agencies including investigation in human participants with a life-threatening condition necessitating urgent interventions (for which available treatments are unsatisfactory), in situations where the participant (or legally authorized representative [LAR]) cannot provide informed consent within the therapeutic window of the investigational product, where there is scientific ground to support that the investigational product will have a prospect of direct benefit to the participant, and there are provisions for seeking informed consent as soon as possible after the intervention. Additional requirements in the US^60,61^ include the need to document that there is no reasonable way to prospectively identify individuals likely to become eligible for participation (i.e., “pre-consent”), public disclosure to and consultation with representatives of the community(ies) in which the research will take place, involvement of an independent data monitoring committee, and having a process for and documentation of attempts to contact a participant’s LAR for consent when possible.

The Working Group recognized the common prior experience with failed enrollment leading to early termination in prior seizure trials,^10,50,64^ ethical concerns regarding the ability to obtain “effective” informed consent under time-sensitive and stressful circumstances,^65^ logistical challenges inherent to neonatal seizure trials (i.e., often conducted at outborn/referral centers where parents are not readily available for in-person consent), and prior reports supporting the general acceptance of EFIC in trials conducted in both adult^66^ and pediatric^67^ studies by patients and families. Nonetheless, there are also reasons that recommendations for EFIC as a standard approach for neonatal seizure trials could be problematic. First, the successful completion of prior acute neuro-intervention trials in the NICU^6,9,56–58^ raises doubt about whether it is justifiable to propose that an investigation could not be practicably carried out without a waiver of consent. Second, the risk for mistrust is a key consideration from the patient/family perspective. While prior studies support that the majority (~70%) of patients and/or families surveyed after participation in a trial enrolled under EFIC were positive about the experience, it is important to note that 15–17% found this unacceptable, with another 10–15% that were neutral or unsure.^66,67^ Third, given the unpredictable nature and variable etiologies of neonatal seizures, particularly seizures from unexpected acute etiologies that begin within hours of birth, it is unclear whether disclosure to and consultation with an “at-risk” community is feasible prior to initiation of a neonatal seizure trial. It was generally agreed that obtaining informed consent with signature(s) from parent(s)/guardian(s) is the preferred approach when enrolling neonates in a clinical trial investigating ASMs, however, there may be certain circumstances where EFIC could be considered. These circumstances would primarily depend upon adequate scientific justification that the therapeutic window for initiation of an investigational ASM (that has strong rationale for prospect of direct benefit to individual participants) would preclude the opportunity to obtain valid or effective informed consent from an LAR, such as for neonatal status epilepticus. Although it is acknowledged that views regarding the ethical permissibility of the use of an EFIC consent paradigm may vary across individuals and institutions,^68^ the justification for any individual ASM trial would need to be reviewed on a case-by-case basis by local institutional review boards (IRBs) and regulatory agencies.

### Approaches to optimizing the informed consent process

The working group reviewed studies reporting feedback on improving the consent process from NICU parents who were approached for participation in a clinical trial, from which they identified several opportunities to optimize the approach to recruitment and enrollment in clinical trials conducted in the NICU. General themes included reducing variability of the consent process across investigators and sites, incorporation of technology, and addressing barriers and disparities (Table 1).

**Table 1:** Recommendations to optimize trial recruitment and consent

1. Reduce variability across investigators and sites
a. Communication skills training and education of investigators
b. Involvement of the clinical team in the consent process
c. Incorporation of study-specific scripts, visual aids, checklists, talking points
2. Incorporate technology
a. Use of interactive electronic-based technology (e.g., diagrams, images, graphics, videos, narration)
b. Video teleconferencing to include both parents during consent discussions
c. Use of electronic informed consent (eIC)
3. Address barriers/disparities
a. Recognize disparities (racial, ethnic, socioeconomic) in research participation
b. Address language barriers via in person or tele-interpreters
c. Address limited medical literacy during the design of the consent process and development of informed consent documents
d. Incorporate assessment tools to test and ensure comprehension and voluntariness
e. Foster awareness about research and potential benefits of participation through community-based education and outreach

Several studies have highlighted the importance of reducing variability across investigative team members in communication and consenting skills.^45,69^ While some studies have found that the role of the person who approached for research participation did not impact enrollment decision,^49^ it was noted that pediatric neurologists, neonatologists, and research staff may benefit from formal communication skills training to ensure an effective and consistent consent process for a medically complex condition such as neonatal seizures. Parental preference for a confident communication style, early approach to allow maximal time for decision-making, and involvement of the clinical team (i.e., individuals who are knowledgeable about the clinical condition and eligible patient’s status) in the consent process has been reported across studies.^45,46,49^ While questions were raised regarding whether the involvement of the clinical team in the consent process may introduce the risk of therapeutic misconception,^70^ it was felt that this could be managed with mitigation of real or perceived conflicts of interest, incorporation of an independent research monitor, and appropriate and precise communication regarding the distinction between clinical care and research interventions. In addition, recommendations for optimizing consistency across individuals involved in the consent process include training and education on communication skills and incorporation of study-specific scripts, checklists, or talking points. Parent advocates and nurses emphasized the importance of family and nursing input not only early in the study design process, but also specifically in the discussion on the optimal approach to the consent process. Finally, interpreters should be used (when feasible with translated consent forms) to ensure effective communication and to optimize equitable enrollment.

Technology provides important strategies to improve intra- and inter-site variability, as well as the overall consent process. Informational video/visual aids can help ensure information delivery is standardized across participating centers and investigators. Several studies have emphasized the importance of involving both parents and ensuring (when possible) that both were approached at the same time for consent.^45,46^ This may be logistically challenging at regional referral hospitals where many trials are conducted and where eligible neonates may be transported without one or both parents. The use of video teleconferencing can be helpful in these situations and should be the preferred method to discuss research participation when neither parent/guardian is available for in-person discussion. The requirements for “wet signatures” on a paper document has also been cited as a burdensome factor that may impede the informed consent process.^69^ The use of electronic informed consent (eIC) may not only alleviate this concern, but can also facilitate the incorporation of interactive electronic-based technology (e.g., diagrams, images, graphics, videos, and narration) into the consent process. While increasing the use of eIC in neonatal trials is recommended, important considerations to highlight include the need for a secure, user-friendly and compliant system, a method to ensure that the person signing is the participant’s LAR, and preservation of a “paper option” to ensure that those without the ability or resources to use eIC technology are not disadvantaged regarding research participation.^71^

Disparities in research participation are well documented and have many contributors, including historical events affecting trust, implicit and explicit biases of investigators, and socioeconomic factors contributing to practical barriers (e.g., financial, transportation, etc.).^48^ While awareness is an important step in addressing disparities, solutions are critically needed. Studies have highlighted the importance of creating more awareness about research and the potential benefits of participation early in pregnancy^46^ as a general approach to improving parental perceptions of research, but this may be even more effective when targeted to underrepresented communities. While potential direct benefits of a particular research trial are necessarily communicated during the study-specific consent process, general benefits to research participation are less often emphasized and may include altruism (i.e., contributions to science and medicine, improving lives of future infants) and potential for optimal monitoring and standardized care that may be afforded with clinical trial participation.^45,72^ These benefits may be most effectively communicated via community-based outreach and education. Addressing limited medical literacy in the consent process is another important strategy, as this, among other factors, can negatively influence comprehension^65,73^ and voluntariness^74^ of informed consent in the emergency setting. A commonly cited challenge to the informed consent process is balancing the inclusion of all essential elements required by research regulations with the potential for overwhelming parents/guardians with the volume of detailed, complex information presented. This concern may be alleviated, although not eliminated, by use of “short” or “modified” consent forms^73^ or the inclusion of “key information” at the beginning of the informed consent document as required by the revised Common Rule in the US.^75^ Other proposed strategies include the incorporation of assessment tools during the consent process to test and ensure understanding and voluntariness.^73,74^

## Trial infrastructure and operational feasibility

While some aspects of trial logistics were addressed in the original publication, the current working group recommended inclusion of important requirements and recommendations for trial logistics and infrastructure. Since seizures are relatively uncommon, occurring in ~1–5/1000 liveborn neonates, multisite trials are needed to enroll sufficient numbers of subjects for all but the most preliminary trials. For multisite neonatal trials, it is essential that the primary coordinating center possesses the regulatory, database, and financial management infrastructure to determine recruitment site feasibility as well as provide comprehensive oversight to ensure compliance with regulatory, database, and monitoring requirements across all participating sites. Identifying sites capable of effectively participating in a trial is crucial and depends on various factors, including the specific trial design (e.g., first-line vs add-on therapy) and the infrastructure and personnel available at each site. It is strongly recommended that trial leaders determine trial sites by using a screening questionnaire. This questionnaire should include the necessary elements of the trial logistics, including availability of appropriate clinical expertise (ideally pediatric neurophysiologists with specific expertise/experience interpreting neonatal EEG), conventional video-EEG capabilities with remote access for 24/7 monitoring, Research Pharmacy/ists capable of managing randomization, ASM/control preparation with masking (if needed), pediatric neuroradiologists and research staff capable of timely data collection and entry for monitoring visits. To help ensure feasibility, each site should estimate the number of eligible neonates with seizures of the etiologies to be included using recent data. Access to electronic medical records, particularly remotely, may be needed for monitoring, as well as the potential for direct downloads of electronic records to import to the research database. The requirements for prolonged, conventional video-EEG monitoring, research pharmacy, and pediatric neurophysiologists with specific expertise/experience interpreting neonatal EEG limit the number and location of institutions that could participate in these trials. Improved access to cvEEG, neurophysiology expertise and ideally automated seizure detection software, would improve the ability to conduct trials in more centers and in low-resource areas with different and/or unique patient populations, as results obtained in trials conducted in high-resource areas may not be extrapolated to other populations.

### Regulatory, database and monitoring requirements

Neonatal seizure trials should adhere to the same rigorous standards for regulatory compliance (such as central IRB), database management, and monitoring, as all clinical trials, to ensure the highest quality and consistency. Particular attention should be given to understanding and adhering to the varying requirements of recruitment sites and country-specific regulatory authorities.

### EEG monitoring

A significant challenge for neonatal seizure treatment trials is the need for high-quality conventional video-EEG monitoring (not aEEG) that can be initiated promptly and monitored remotely in real time by expert EEG readers. This requirement necessarily limits the hospitals in which seizure treatment trials can be conducted, since 24/7 EEG placement and monitoring for neonates is by no means universally available, even at many tertiary care level institutions, and remains a challenge for clinical trials.^76^ Most importantly, there is a need for 24/7 availability of expert readers, i.e., pediatric neurophysiologists (ideally) with experience interpreting neonatal EEG. Seizure detection algorithms for automated seizure detection would likely be the most efficient and cost-effective approach to seizure detection (and analysis) and estimation of seizure burden. Neonatal seizure detection algorithms are now commercially available and may be a valuable tool in neonatal seizure trials, with the potential to reduce time to seizure recognition and treatment. However, due to the frequent occurrence of artifacts in neonatal EEGs, these algorithms alone are not sufficiently reliable. Instead, they serve as assistive tools, with human experts remaining essential for making diagnostic and treatment decisions. Furthermore, central real-time EEG review is challenging because of costs and institutional limitations on remote EEG access and privacy; central review by EEG technologists using a commercial EEG monitoring company was not sufficient without investigator involvement in one trial.^76^ In summary, conventional video-EEG monitoring data should be monitored ideally in real time by expert neonatal EEG readers, with subsequent masked central review of EEG data for final analyses, at least until there is available and robust seizure detection/analysis software.

### Data and safety monitoring board (DSMB)/steering committee

As described in the original publication, an external DSMB should contain appropriate expertise, including Neonatal/Pediatric Neurology, Neurophysiology, Neonatology with expertise in neonatal seizures and associated etiologies, and a statistician with trial experience. A family stakeholder representing patient families of children who experienced neonatal seizures may be included to provide the patient family perspective on the DSMB, depending on local IRB requirements. Similarly, some trials will benefit from guidance from a Steering Committee that may include the senior investigators on the trial, as well as external experts who can provide guidance regarding trial design, conduct, and analyses, particularly regarding any challenges that arise during trial conduct that might require modification of aspects of trial design or analyses.

## Summary

In summary, while the original recommendations for neonatal seizure trials remain largely supported in this updated version, the Working Group updated and added to the original recommendations based on recent experiences from several trials and developments in the field. The trial design recommendations were not substantially changed, particularly given the lack of new data to inform definitions of optimal efficacy endpoints, but the Working Group recommended inclusion of alternate measures of seizure burden reduction as secondary or exploratory endpoints, to help elucidate clinically meaningful efficacy endpoints for use in future trials. The potential for unmasked or single-masked trials was discussed, given practical issues related to adverse effects of phenobarbital, the standard comparator. Additional details have been provided regarding recommended reporting of concomitant medications and AEs. Key covariates to incorporate into the study design were described in the first paper, but other important covariates should also be reported and analyzed, such as timing of seizure onset/cessation, randomization, and ASM administration. New recommendations regarding genetic testing were provided; this aspect of neonatal seizure management and trial design will likely continue to evolve rapidly. Importantly, many specific recommendations were added regarding improved strategies for recruitment and consent, including the use of novel technologies and the involvement of patient advocacy groups. Recommendations regarding trial infrastructure and operational feasibility were included to facilitate trial initiation and conduct, given the many logistical challenges of conducting neonatal seizure treatment trials. Finally, these recommendations take into account some accommodations for local or national regulations and resources, to ensure that trials are conducted as appropriate to the setting in which the patients are treated.
